# Aromatic Ester Bioplastics from Wood and Cellulose: Cinnamates as Greener Alternatives to Benzoates

**DOI:** 10.3390/ma19030574

**Published:** 2026-02-02

**Authors:** Luke Froment, Jacqueline Lease, Prabu Satria Sejati, Firmin Obounou Akong, Christine Gérardin Charbonnier, Yoshito Andou, Philippe Gérardin

**Affiliations:** 1 Laboratoire d’Etudes et de Recherche sur le Matériau Bois (LERMAB), French National Research Institute for Agriculture, Food and Environment (INRAE), Université de Lorraine, 54000 Nancy, France; luke.froment@univ-lorraine.fr (L.F.); christine.gerardin@univ-lorraine.fr (C.G.C.); 2 Graduate School of Life Sciences and Systems Engineering, Kyushu Institute of Technology, Kitakyushu 808-0196, Japan; lease.jacqueline285@mail.kyutech.jp; 3 Research Center for Biomass and Bioproducts, National Research and Innovation Agency (BRIN), Tangerang Selatan 15314, Indonesia; prab004@brin.go.id

**Keywords:** bioplastic, thermoplastic, esterification, pine sawdust, microcrystalline cellulose, cinnamate, TFAA, π–π stacking, mechanical properties, hydrophobicity

## Abstract

**Highlights:**

**What are the main findings?**

- Lignocellulose benzoates and cinnamates are thermoplastic.
- The pine esters had better properties than the MCC esters.
- The cinnamates had improved properties compared to benzoates.

**What are the implications of the main findings?**

- Wood cinnamate esters are a superior alternative to cellulose benzoates.
- These materials could potentially be used as bioplastics for packaging.

**Abstract:**

To address the environmental impact of petroleum-derived plastics, lignocellulose esters provide a promising renewable alternative. However, research has primarily focused on linear cellulose esters, leaving raw biomass aromatic derivatives largely overlooked. Herein, we report a one-pot, room-temperature synthesis of cinnamate and benzoate esters from microcrystalline cellulose (MCC) and raw pine sawdust. A breakthrough finding reveals that pine esters consistently outperform pure MCC, achieving tensile strengths of 5–8 MPa (vs. 1–3 MPa for MCC) possibly due to a lignin-driven synergistic effect facilitating π–π stacking. The resulting films are hydrophobic (contact angles 80–100°) and fully thermoplastic. Cinnamates emerge as a technically superior and “greener” alternative to benzoates, paving the way for the direct upcycling of wood waste into sustainable packaging materials within a circular economy.

## 1. Introduction

Plastics are the third most produced type of material in the world, behind steel and concrete [1,2,3]. The overwhelming majority of these plastics are manufactured from fossil resources [4,5], and their production contributes significantly to climate change through substantial emissions of carbon dioxide [6,7]. Plastics derived from plant biomass have therefore received considerable attention as potential renewable alternatives with a lower carbon footprint. However, the current major bioplastic, polylactic acid (PLA), is industrially produced from edible crops like corn and sugarcane, which require large amounts of fertilizers and water [8,9,10]. To improve sustainability, attempts have been made to produce “second-generation” bioplastics from lignocellulosic by-products, such as sawdust from the wood industry, which is currently mostly used for energy [11].

Wood is primarily composed of three biopolymers: cellulose, hemicelluloses, and lignin. Inducing thermoplasticity in cellulose often involves chemical modification, most commonly through the esterification of hydroxy groups [12]. Traditional acylation methods frequently rely on acyl chlorides or anhydrides, which can be expensive, moisture-sensitive, and require toxic solvents or bases for neutralization [13,14,15,16,17,18,19]. To address these challenges, our group has previously developed a one-pot method for wood esterification using trifluoroacetic acid anhydride (TFAA) as an impelling agent [20].

**Figure 1:**
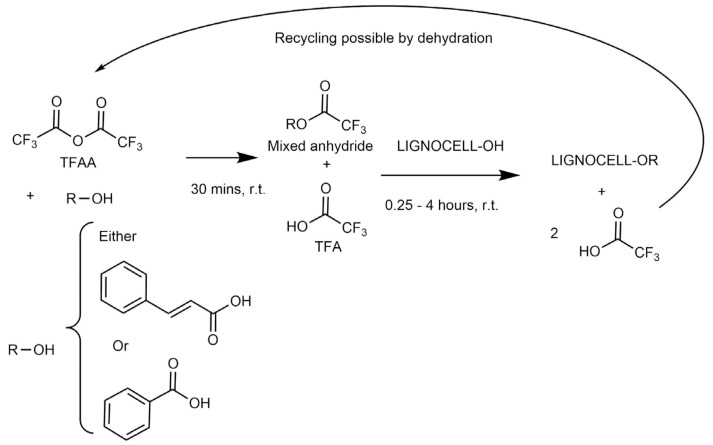
Overview of the TFAA/TFA system applied to aromatics.

This system operates at room temperature and atmospheric pressure, allowing for a simplified process where the TFA/TFAA can be recovered and recycled in a closed loop [21,22,23] (Figure 1). Recent reviews and experimental studies have confirmed that, apart from its acidity, TFA is of low environmental concern and is biodegradable [24,25,26,27,28,29].

While linear cellulose esters have been extensively researched, aromatic esters of lignocellulosic biomass, such as wood, have received considerably less attention [30,31,32,33,34,35,36]. Bioplastics polyesters produced by microbial fermentation have recently been shown to have different behavior to their linear counterparts [37], it would be interesting to investigate whether this is also the case for lignocellulose esters. Previous studies on wood benzoylation or cinnamoylation focused primarily on photo-stabilization rather than inducing thermoplasticity [38,39,40,41]. Furthermore, benzoic acid is increasingly scrutinized due to its classification as a health hazard [42]. Cinnamic acid additionally has the advantage of a lower acute toxicity as shown by its median lethal dose (LD50) of 2 g/kg body weight in rats by oral route compared with 1.7 g/kg for benzoic acid [43,44]. In this context, cinnamic acid emerges as a “greener” and safer alternative that can be more readily obtained from biomass. To the best of our knowledge, no previous attempt to produce a thermoplastic material by esterifying lignocellulose with cinnamic acid has been reported. Our goal is therefore to extend this method to synthesize and fully characterize novel, bulky aromatic esters from both pure cellulose and raw wood waste, evaluating their thermal and mechanical properties as potential sustainable alternatives to fossil-based plastics.

## 2. Materials and Methods

Cinnamic and benzoic acid (both from Merck/Sigma Aldrich (Darmstadt, Germany/St. Louis, MO, USA), synthesis grade > 99% pure, Saint-Quentin-Fallavier, France) and trifluoroacetic anhydride (TFAA) from Thermo-Scientific (purity ≥ 99%, Villebon-sur-Yvette, France) were used. Pine sapwood (*Pinus sylvestris*) was obtained from Poirot Construction Bois (La Bresse, France). The wood material was ground, sieved to fine sawdust (120 mesh), Soxhlet extracted using a mixture of toluene and ethanol (1:2 *v*/*v*) for 4 h, followed by ethanol for another 4 h to obtain extract free sawdust. Microcrystalline cellulose (MCC) powder with an average particle size of ~20 µm was acquired from Sigma-Aldrich (Tokyo, Japan). The pine sawdust and MCC were kept in a dried state in an oven at 103 °C overnight before use.

### 2.1. Preparation of Lignocellulose Derivatives

An equimolar amount of aromatic (either cinnamic or benzoic) acid and TFAA was mixed in a glass reactor. The reactor was sealed, and the mixture was left at room temperature without stirring. Thirty minutes after the formation of a homogeneous phase, an initial mass m0 of substrate (MCC or sawdust) was added to the mixed anhydride in the reactor. The reaction was allowed to proceed at room temperature and was then quenched with water. The solid was filtered on a Buchner funnel under vacuum and washed with ethanol and water. Final purification of the esterified material (MCC or sawdust) was carried out by Soxhlet extraction overnight with a 2:1 *v*/*v* ethanol:water mixture. The purified solid was then left to dry under a fume hood for a day (24 h) and then dried at 103 °C until a constant mass was achieved (typically 1–2 h), which was recorded as the final mass m1. The mass ratio of TFAA to pine sawdust for the cinnamic acid esterification was varied from 1:1, 3:1, 4:1, and 8:1 g/g, and for the 4:1 ratio, the reaction time was varied from 15 min, 1 h, 2 h, and 4 h. The optimized conditions were used for the other three combinations (benzoic acid and/or MCC).

The Weight Percent Gain (WPG in units of %) was calculated from the initial (m0) and final dry masses (m1), according to Equation (1) [45]:

WPG = 100 × (m1 − m0)/m0(1)

Ester content, E. C., (in mmol/g of substrate) was calculated from the WPG and molar mass of the acid (M) using equation as follow (Equation (2)) [45]:

E. C. = (1000/100) × WPG/(M − 18) = 10 × WPG/(M − 18)(2)

The reactions with optimized conditions were performed in duplicate, with 3 g of substrate each time which enabling the calculation of standard deviations.

### 2.2. Material Characterization

Fourier transform infrared (FTIR) spectroscopy was used to examine the chemical structures of the MCC and pine sawdust esters, using a Nicolet iS5 spectrometer (Thermo Fisher, Waltham, MA, USA). The Attenuated Total Reflectance (ATR) method was utilized for this analysis. All spectra were recorded within the range of 4000 to 650 cm^−1^, with a resolution of 4 cm^−1^, accumulating 32 scans.

Solid-state CP/MAS 13C NMR spectra were recorded using CPMAS BL7 probes (Bruker Corporation, Bruker, MA, USA) at a 13C frequency of 75.47 MHz (superconducting magnet with 7.05 Tesla effective field) on a Bruker MSL300 spectrometer (Bruker Corporation) with a 5 kHz spinning rate. The acquisition time was 0.026 s with ~1200 transients. The relaxation delay was 5 s, Cross Polarization time was 1 ms, and spectral width was 20,000 Hz.

Powder XRD patterns were collected using a Rigaku MiniFlex 600 diffractometer (Rigaku Analytical Devices, Tokyo, Japan) equipped with a sealed Cu tube operated at 30 kV and 15 mA and a D/teX Ultra 1D silicon strip detector. The instrument was configured in Bragg–Brentano reflection geometry with fixed optics: a 0.625° divergence slit, 5° incident and receiving Soller slits, a 1.25° anti-scatter slit, and a 0.3 mm receiving slit. Samples were mounted on a zero-background silicon sample holder and spun at approximately 30 rpm during data collection to minimize preferred orientation effects. Data were collected over the 3–70° 2θ range using a continuous scan with a step size equivalent to 0.02° and a scan speed of 10°/min, resulting in a total scan duration of approximately 7 min. The radiation source was Cu Kα with a weighted average wavelength of λ = 1.5418 Å, obtained after Kβ suppression with a Ni filter. Both Kα_1_ (1.5406 Å) and Kα_2_ (1.5443 Å) components were present.

The powder patterns were analyzed using OriginPro 2022b (OriginLab, Northampton, MA, USA) with pseudo-Voigt peak functions. A linear background subtraction was applied. The crystalline reflections were fitted using pseudo-Voigt profiles, while the amorphous contribution was modeled as a broad peak centered near 18.6° 2θ (the minimum between the 110 and 200 reflections of cellulose Iβ). The crystallinity index (CrI%) was calculated using two approaches:

(i) Peak deconvolution method: CrI was determined from integrated areas according to [Equation (3)]:

CrI (%) = (Acrystalline/(Acrystalline + Aamorphous)) × 100(3)

where Acrystalline is the summed area of the deconvoluted crystalline peaks and Aamorphous is the area of the fitted amorphous contribution.

(ii) Segal method [46]: CrI was calculated as [Equation (4)]:

CrI (%) = ((I200 − IAM)/I200) × 100(4)

where I200 is the maximum intensity of the (200) reflection near 22–23° 2θ, and IAM is the minimum intensity between the (110) and (200) reflections.

The spacing between layers, d in a crystal was calculated using Bragg’s law with the [Equation (5)]:

n λ = 2 d sin θ(5)

where n is the reflection order, λ is the wavelength of the X-ray beam, and θ is the angle between the lattice planes and the incident beam.

Thermal gravimetric analysis (TGA) was performed using a TGA/DSC 1 LF/1100 (Mettler Toledo, Greifensee, Switzerland) equipped with a MultiSTAR^®^ six thermocouple ceramic sensor which was connected to a Windows 10 PC running the STARe V.14 program. For each sample, ~10 mg of sample was placed in a 70 μL aluminum oxide pan. Heating was supplied from 30 up to 600 °C at a heating rate of 10 °C/min under a nitrogen atmosphere (50 mL/min).

Differential Scanning Calorimetry (DSC) measurements were obtained using a DSC 1/700 instrument (Mettler Toledo, Greifensee, Switzerland) equipped with a 120 Au/Pd thermocouple ceramic High Sensitivity Sensor (HSS8) which was connected to the same PC. Samples of ~10 mg were placed in 40 μL aluminum pans with covers, and a nitrogen flow of 50 mL/min was used. A heat-cool-heat procedure was used, with heating from 30 to 150 °C, 1 min isotherm, cooling to 30 °C then a second heating from 30 to 450 °C, with a rate of 10 °C/min.

Thermomechanical analysis (TMA) was performed using a TMA/SDTA 2+ LF/1100 (Mettler Toledo, Greifensee, Switzerland) connected to the same PC. Samples were cut according to manufacturer specifications (~10 × 2 × 1 mm) and placed on a quartz support. Analysis was performed in a 3 point-bending configuration with heating from 30 to 310 °C at a rate of 10 °C/min, a force of 0.1 N and a nitrogen flow rate of 50 mL/min.

The contact angles of lignocellulose derivative films were assessed by photographing small droplets of water on the film samples using a contact angle meter (DMs-401, Kyowa Electronic Instruments Co., Ltd., Tokyo, Japan). Five droplets of deionized water were placed on the surface of the lignocellulose derivative films. To account for surface heterogeneity, readings were taken on each sample and averaged.

For mechanical testing, samples were pressed at 180 °C under a pressure of 20 MPa for 20 min using a hot press machine (HC300-15, As One Corporation, Osaka, Japan). Subsequently, the samples were cut into dog-bone shapes according to the JIS K-7113-2 [47] standard dimensions (115 mm × 25 mm) prior to testing. The mechanical properties of each film were elucidated using a tensile and compression machine (Minebea Mitsumi Inc., Tokyo, Japan). Three key parameters were measured: Young’s modulus, tensile strength, and elongation at break. The tests were conducted with a 1 kN load cell and a crosshead speed of 10 mm/min. Each sample underwent testing in triplicate, with the standard deviation being reported. A PG-01 thickness gauge (Teclock Corporation, Nagano, Japan) was employed to measure film thickness.

## 3. Results & Discussion

In this work, the acid and TFAA are combined to form a mixed anhydride, as well as TFA and in the second step, the hydroxy groups of the substrate react with the mixed anhydride to produce the desired ester as well as unwanted trifluoroacetate. The trifluoroacetate and any unreacted starting products can be removed using a water and ethanol mixture. A considerable advantage compared to other methods is that this reaction proceeds at room temperature, atmospheric pressure, and does not require an inert atmosphere or any base. Trifluoroacetic acid can be recycled back into the anhydride and re-used in a closed-loop process, thereby reducing costs further. The chemical structures, thermal stabilities, and mechanical properties of lignocellulose derivatives were elucidated using different substrates.

### 3.1. Reactivity of Cinnamic Acid

**Figure 2:**
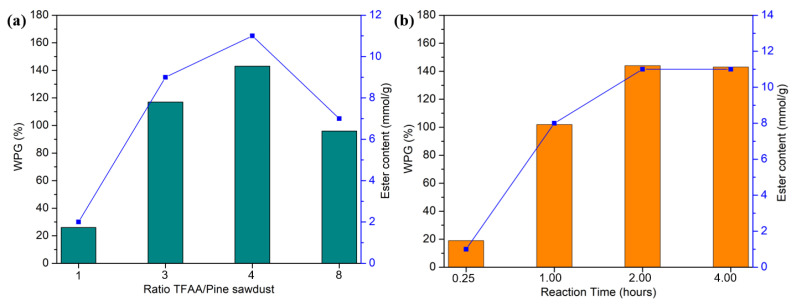
The graph of (**a**) WPG and ester content at different TFAA/Pine ratios, and (**b**) WPG and ester content over various reaction times for a ratio TFAA/Pine of 4:1.

The variation in reactant ratios and reaction times allowed for the determination of optimal conditions for the pine cinnamate, based on WPG: a mass ratio of TFAA to sawdust of 4:1 g/g and a reaction time of 2 h (Figure 2). This confirmed that the reactivity of this acid was similar to what was observed for linear acids, with optimal conditions of 4 h and a 4:1 ratio [35]. The decrease in WPG and ester content when the ratio was increased from 4:1 to 8:1 could be due to degradation caused by the larger amounts of TFA by-product.

The 4:1 ratio and 4 h conditions were then also used for the MCC and benzoates to allow us to study the effect of the substrates and of the aromatic acid. These conditions can then enable comparisons with results from previously published studies with linear acids.

### 3.2. WPG and E.C of the Esterified Lignocellulose

**Figure 3:**
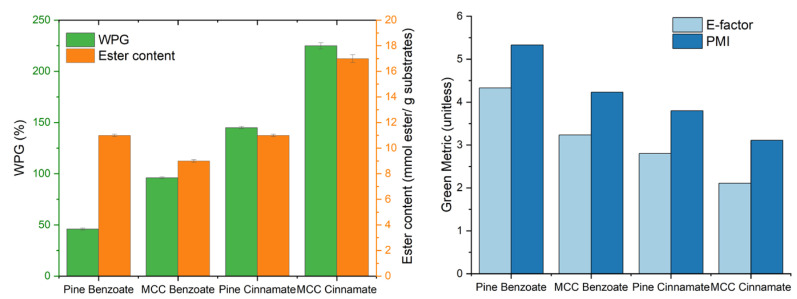
WPG and ester content of benzoates and cinnamates with different substrates (**left**) and the green metrics values (**right**).

Figure 3 displays the WPG and ester contents of Pine Benzoate, MCC Benzoate, Pine Cinnamate and MCC Cinnamate. The WPG and ester contents indicated that more ester was grafted onto the MCC than onto the pine sawdust. This could potentially be explained by the fact that cellulose has more hydroxy groups per monomeric unit than hemicelluloses, which in turn have more hydroxy groups than lignin [48] and that pine sawdust is a mixture of the three.

This explanation is also consistent with our previous study where esterified spruce sawdust and its separated biopolymers with linear myristic acid (C14). The WPG and ester contents followed the trend: cellulose > sawdust > lignin > hemicelluloses [32]. The cinnamates exhibited higher WPG compared to the benzoates, which was expected due to their higher molar mass. Furthermore, the cinnamates had higher ester content than the benzoates, suggesting that the cinnamic mixed anhydride reacted more efficiently. This trend is similar to that observed using the SolReact method (aqueous suspension, 130 °C, 20 h) applied to cellulose nanocrystals. The amounts grafted with our method for the benzoate is significantly higher, compared to the SolReact value which was close to zero [42]. Additionally, the WPG values for the pine esters are higher than those obtained using vinyl esters [41], demonstrating the versatility and superiority of our method. These data can be used to estimate the greenness of the reactions by simple mass-based green metrics. The most commonly used are the Environmental factor (E-factor, the ratio of mass of waste and mass of product) and the Process Mass Intensity (PMI, the ratio of the starting reagents and mass of product) [49]. The calculated average values in Figure 3 show that the cinnamate and benzoate esters are closer to the ideal values of 0 for the E-factor and 1 for the PMI. The cinnamates are therefore ‘greener’ than the benzoates when produced by our method.

### 3.3. Chemical Characterization of Esterified Lignocellulose

**Figure 4:**
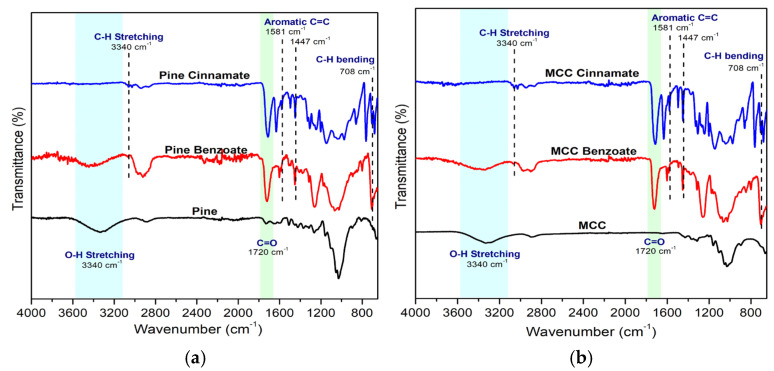
FTIR spectra of (**a**) native pine and esterified pine, and (**b**) native Microcrystalline Cellulose (MCC) and esterified MCC.

Figure 4 shows the FTIR spectra for lignocellulose, including MCC and pine sawdust, as well as their modified products. The FTIR spectrum of MCC esters and pine esters showed the disappearance of the peak at 3450 cm^−1^, corresponding to O–H stretching, after esterification. For pine cinnamate, pine benzoate, MCC cinnamate, and MCC benzoate, peaks are observed at 708 cm^−1^, 1581 cm^−1^, 1447 cm^−1^, and 1720 cm^−1^, indicating out-of-plane aromatic C–H bending, C=C stretching, and C=O stretching, respectively [49]. The carbonyl peaks, C=O, confirmed that surface hydroxy groups have reacted with cinnamic acid and benzoic acid as acylating agents, successfully grafting the aromatic rings onto lignocellulosic materials [50,51].

**Figure 5:**
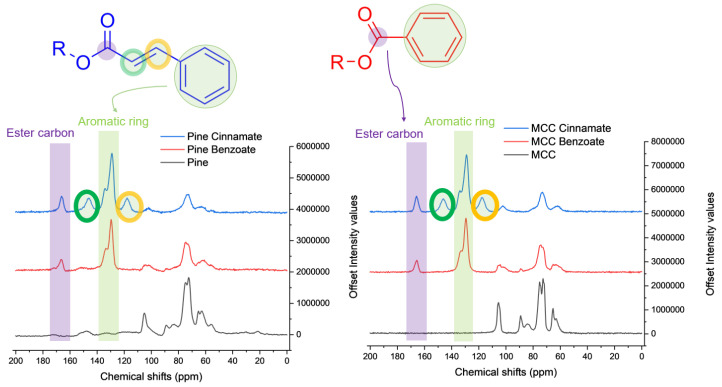
13C NMR spectra of native and esterified pine (**left**) and native and esterified MCC (**right**).

The NMR spectra in Figure 5 confirm that aromatic groups have been grafted with characteristic peaks at ~130 ppm corresponding to the carbon atoms of the aromatic ring and ~170 ppm corresponding to the ester carbon with an aromatic substituent. Native MCC had no peaks nor multiplets in that range but native pine did have some weak and broad signals due to the lignin. The peaks in the 50 to 110 ppm range are mainly due to the polysaccharide carbons. The cinnamate esters have two additional peaks at 120 and 150 ppm due to each of the two carbon atoms in the double bond between the carbonyl and aromatic moieties.

### 3.4. Structural Properties of Esterified Lignocellulose

**Figure 6:**
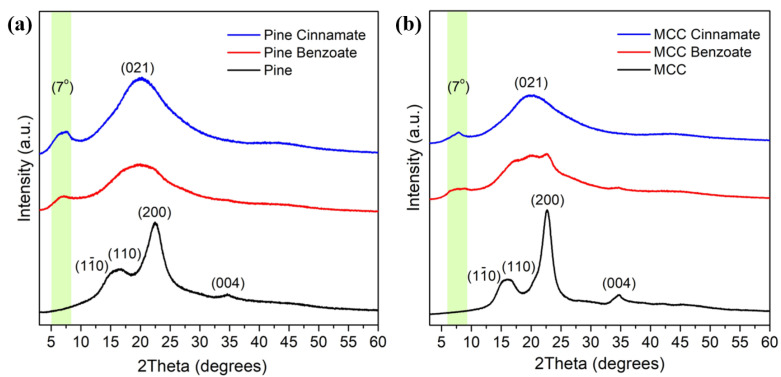
XRD patterns of (**a**) native pine and esterified pine, and (**b**) native MCC and esterified MCC.

XRD patterns of the initial MCC and pine samples are shown in Figure 6. The well-known diffraction peaks at 2θ around 14.9°, 16.3°, 22.5°, and 34.6° were assigned to the ($\overset{-}{110}$), (110), (200), and (004) planes, respectively, in agreement with the characteristic diffraction pattern of cellulose Iβ.

Following esterification, significant broadening was observed for the (200) and (004) peaks, while an increase in intensity was noted at the (021) plane. This behavior is attributed to the increased presence of amorphous regions caused by the disruption of ordered cellulose chains by the aromatic ester groups. These findings suggest that esterification increased the proportion of disordered cellulose, and the broadening of the diffraction peaks indicates notable structural changes within the cellulose material [52].

**Figure 7:**
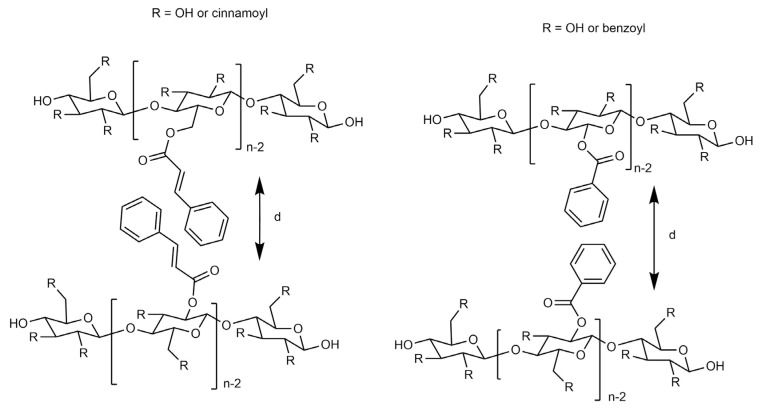
Representation of the esters with the side chains in planar conformation, perpendicular to the side chain planes with or without interdigitation of the alkyl and aromatic side chains.

Additionally, the reflection observed at low Bragg angles has been attributed to the diffraction of planes formed by cellulosic and pine backbones, with their side chains oriented perpendicularly to the planar structure (Figure 7) [53,54]. To assess the relevance of this structural model for the current lignocellulose derivatives, the measured layer spacing was used to infer the arrangement of the side chains.

Pine Cinnamate and MCC Cinnamate exhibited lower layer spacing values compared to Pine Benzoate and MCC Benzoate, despite having longer alkyl chains (Table 1). This outcome suggests the interdigitation of the side chains and aromatic rings due to Pi-stacking between aromatic nuclei and double bonds. The shorter benzoate side chains restrict flexible movement, resulting in interdigitation for MCC benzoate and pine benzoate [55]. In contrast, the flexible cinnamate alkyl chains potentially allow for interdigitation, as indicated by the layer spacing values of 12.94 and 11.19 Å.

The crystallinity index (CrI) of cellulose in the samples was estimated using both the Segal peak height method (Figure S1) and the peak deconvolution method. The results indicate that the crystallinity generally decreased after esterification, reflecting disruption of the ordered cellulose structure (Table 2). For samples such as MCC Cinnamate, Pine Benzoate, and MCC Benzoate, the CrI could not be reliably calculated using the Segal method because the (200) peak shifted after esterification, making the peak height approach unsuitable. However, the peak deconvolution method could still be applied to these samples by fitting the crystalline peaks and the amorphous background, providing a more accurate estimate of the crystallinity changes induced by the esterification (Figures S2–S7). These observations suggest that the introduction of ester groups increased the proportion of disordered regions within the cellulose, consistent with the broadening of the diffraction peaks observed in the XRD patterns.

The starting MCC had higher crystallinity than the wood, which was expected; however, its value is somewhat low since MCC is typically produced by acid hydrolysis of the amorphous parts of cellulose which leave almost exclusively crystalline parts. This lower value than expected can be explained by the fact that the Segal method is not the most accurate method for absolute values; however, it is the simplest and the most commonly used [56,57]. Our pure MCC and pine values are similar to what has been previously published using the Segal method, with reported values of 79 and 56.4% respectively [58,59]. The use of the Segal method remains useful for the relative changes and allows comparisons to literature data. The crystallinity indices are significantly lower after the modification process which explains the high WPG and ester content values and is consistent with previously observed behavior for linear acids, where it was found that the polar TFA decrystallizes the cellulose [35].

### 3.5. Thermal Properties of Esterified Lignocellulose

**Figure 8:**
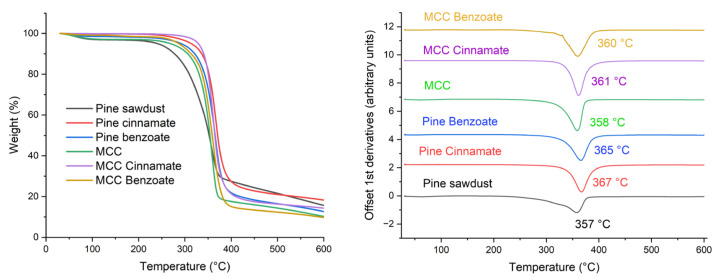
TGA curves of native pine and MCC as well as the esterified lignocellulose (**left**) and offset DTGA curves with Tmax peak temperatures (**right**).

The thermal properties of both unmodified and modified powders were analyzed using thermogravimetric analysis (TGA) to assess their thermal transitions and degradation under a nitrogen atmosphere. As shown in Table 3 and Figure 8, the thermal degradation (Td) of native pine commenced at 313 °C. In contrast, Pine Benzoate and MCC Benzoate began decomposing at 339 and 345 °C, respectively, indicating an enhancement in thermal stability due to benzoylation and cinnamoylation [60].

Similarly, pure MCC and its modified forms exhibited comparable trends. The onset and maximum degradation temperatures for MCC were observed at 333 and 358 °C, respectively. For MCC Benzoate and MCC Cinnamate, initial decomposition temperatures (Tonset) were recorded at 333 and 342 °C, with maximum degradation temperatures (Tmax, peak of the first derivative) reaching 360 and 361 °C. These findings demonstrated that modified cellulose and pine sawdust possessed superior thermal stability compared to unmodified materials. The hydrophobic modifications have altered the crystalline regions, increased the amorphous character and consequently enhancing the thermal stability of pine sawdust and MCC [61,62]. This is an advantage compared to some other modification methods, which have led to a decrease in thermal stability [63,64]. The TGA curves in Figure 8 show that the cinnamates have higher residual weight than the benzoates at the end (600 °C). This could be explained by the cinnamic moiety having a higher proportion of carbon atoms to oxygen atoms (C9:O2) than the benzoic moiety (C7:O2) and therefore potentially forming more char. The pine sawdust and its esters have a larger amount of residue than the MCC and its respective esters, which can be explained by the lignin that is present in the wood, which is well known to char more readily than cellulose.

**Figure 9:**
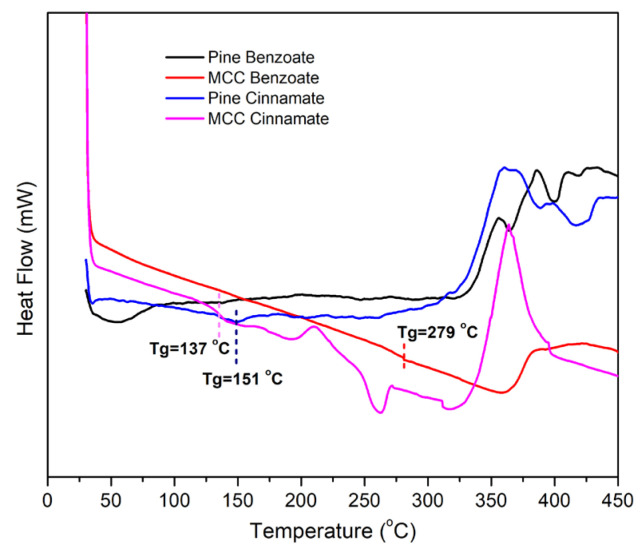
DSC thermograms of modified pine and MCC.

The DSC results of modified pine and MCC, which are presented in Figure 9 and Table 4, confirm the degradation behaviour and provided additional information. These results suggested possible glass transition temperatures (Tg) for the MCC Benzoate, MCC Cinnamate and Pine Cinnamate which are 279, 137, and 151 °C respectively. Analyzing the results for pine wood is more challenging because wood is a mixture of three components: cellulose, hemicelluloses, and lignin. The modified pine samples had a maximal exotherm at 300–450 °C which is in the range of the known thermal decomposition of the lignocellulose [65].

In addition, the determination of softening temperatures (Ts) using TMA is crucial as it can provide insights into the thermal behavior of materials. The initial softening observed in the TMA results can be attributed to the side chains because they tend to have lower molecular weights and are less cross-linked than the main polymer backbone. This makes them more mobile and likely to undergo softening at lower temperatures. In contrast, the secondary softening, attributed to the backbones, occurs at higher temperatures due to the stronger intermolecular forces.

**Figure 10:**
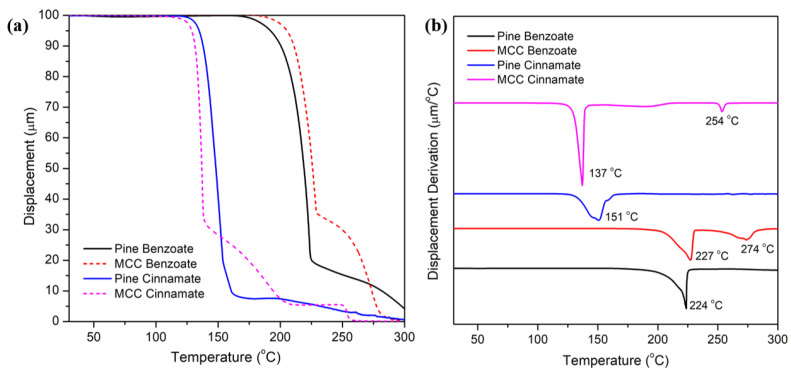
TMA (**a**) and DTMA (**b**) graphs of modified pine and MCC.

In the case of the MCC esters, the presence of two softening points suggested the existence of distinct molecular structures within the material. MCC, being more refined and having a higher degree of purity, exhibited this dual-phase behavior due to the varying interactions of cellulose chains and their crystalline or amorphous regions. Moreover, the pine esters showed only one softening point (Figure 10), likely due to their more complex composition. The diverse components and their interactions could result in a more gradual and less distinct softening process, making it difficult to detect separate transitions. The softening point may correspond to the glass transition temperature since for the samples where a Tg could be detected by DSC the values are close (Table 4). It is known that TMA can be more sensitive to certain thermal transitions such as Tg than DSC. Therefore, the softening temperature of 224 °C for pine benzoate could also be its Tg (which was not detected by DSC). These values therefore allow us to calculated potential processing windows (defined as Tonset − Tg) which are shown in Table 5. As a result of their lower softening temperatures the cinnamates have wider processing windows than the benzoates. This should therefore make them easier to process and shape into rigid packaging.

The presence of the cinnamoyl group, with its conjugated double bonds, could make the polymer chain more flexible and reduce the energy required to initiate softening. In contrast, the benzoate esters had a more rigid structure, which increased the intermolecular forces, and the temperature required for softening. This lower softening temperature of cinnamate esters is consistent with their ability to melt and form films more easily in the previous section.

### 3.6. Surface Morphologies of Esterified Lignocellulose

**Figure 11:**
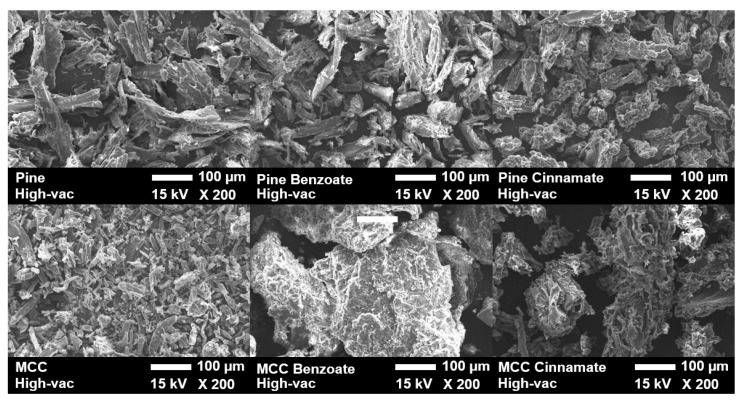
SEM images of unmodified and modified substrate powder at 200× magnification.

**Figure 12:**
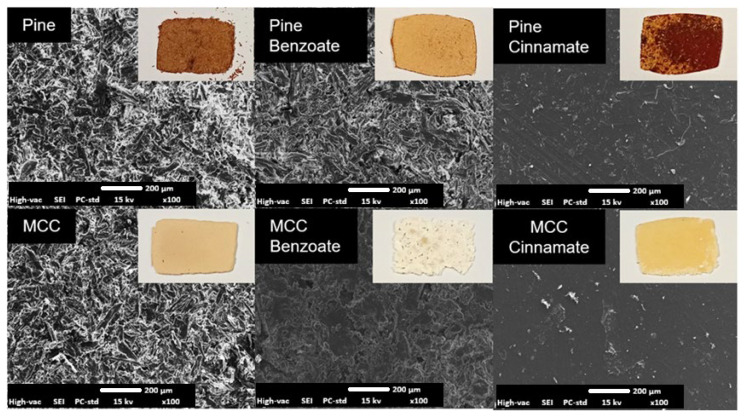
Digital and SEM images of unmodified and modified substrate films at 100× magnification.

The plastic behaviour of the material is demonstrated by the scanning electron microscopy (SEM) images of the unmodified and modified substrates, as well as of their films, shown in Figure 11 and Figure 12.

Before hot pressing, both modified and unmodified substrates appeared quite similar as particles in the micrographs. However, after hot pressing, the cinnamate and benzoate esters formed smoother films compared to the unmodified substrates, which remained rough and unmelted because they were merely compressed together. The higher the ester content is, the smoother the surface is. This difference was also noticeable at the macro level due to color: the cellulose esters appeared whiter and paler, while the pine esters exhibited a browner hue. This coloration was attributed to the presence of lignin in pine, which acted as a chromophore [66,67].

### 3.7. Wettability of Esterified Lignocellulose

**Figure 13:**
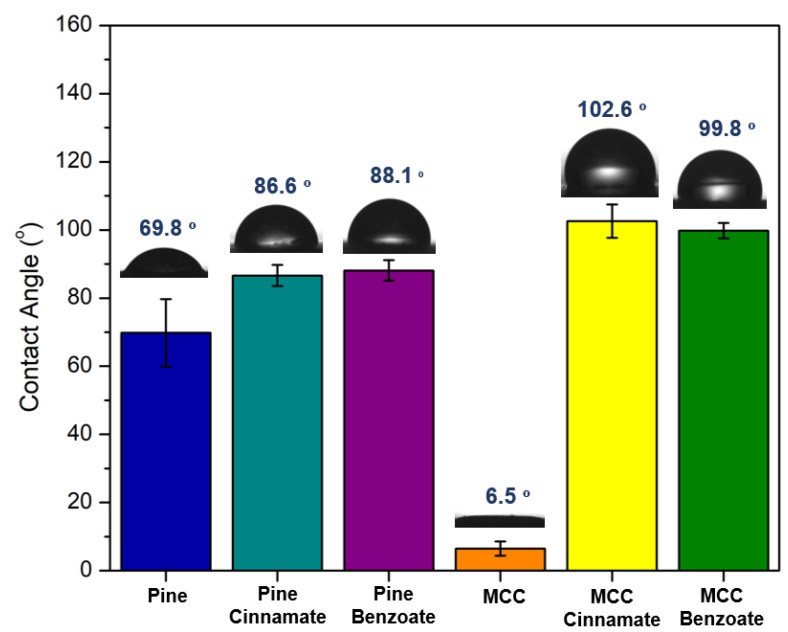
Water contact angle test of modified and unmodified Pine and MCC.

As illustrated in Figure 13, the surfaces of pine and MCC esters exhibited a wettability conversion from hydrophilic to hydrophobic.

The contact angles of water droplets on the surfaces of the films were stable for all of the modified films but for unmodified sawdust, it decreased over time as the water droplet was slowly absorbed by the hydrophilic material (Figure S8). The microcrystalline cellulose absorbed the droplets even faster, in only a few seconds. The modified films had contact angles in the 80 to 100 ° range and therefore can be considered hydrophobic, similarly to previously reported cellulose esters [68]. These values are higher than those reported for polyethylene terephthalate (PET) and polylactic acid (PLA) [69,70].

### 3.8. Mechanical Properties of Esterified Lignocellulose

**Figure 14:**
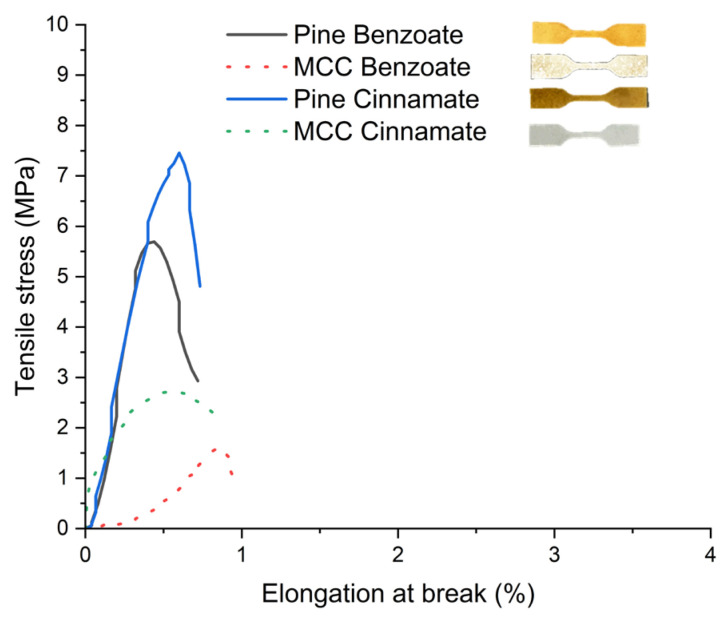
The graph of stress-strain curves of modified pine and MCC.

Although cellulose cinnamate esters have been reportedly synthesized using the TFAA method previously, the materials were generally not fully purified, and no data on their mechanical properties [36] were provided. This study addresses this gap by providing a rigorous assessment of the tensile performance of our novel pine and microcrystalline cellulose (MCC) esters (Figure 14 and Table 6).

A key finding is the consistent superiority of pine esters over their MCC counterparts. Pine cinnamate exhibited the highest tensile strength at approximately 7.86 MPa, nearly three times the value recorded for MCC cinnamate (2.63 MPa). A similar trend was observed for the benzoates, where the pine ester (5.47 MPa) significantly outperformed the MCC benzoate (1.27 MPa). These tensile strength values fall within the ranges previously reported for linear saturated cellulose esters and unsaturated cellulose esters [53,54,71].

This enhanced performance of raw lignocellulose could be attributed to a synergistic effect within the natural wood matrix. Unlike pure MCC, pine contains lignin, whose aromatic structures might engage in π–π stacking interactions with the grafted cinnamoyl or benzoyl moieties. This configuration may promote better molecular cohesion, thereby reinforcing the rigidity and resistance of the final material. This explanation is consistent with previous studies where pi–pi stacking between the aromatic groups of different lignin molecules within composites was found to lead to improved properties [72,73]. Furthermore, the cinnamate esters displayed superior mechanical properties compared to the benzoates (3–8 MPa vs. 1–5 MPa), likely because the cinnamate group can more easily adopt a favorable configuration for stacking with the aromatic parts of lignin. Additionally, the higher degree of polymerization of cellulose present in pine sawdust comparatively to that of MCC could also be one factor that potentially plays a role in these enhanced performances [74,75]. While these materials exhibit high stiffness and the inherent brittleness typical of unplasticized aromatic esters with elongation at break values remaining below 1% their values are comparable to those of PLA [76], although the tensile strength of PLA is higher. Specifically, the pine cinnamate tensile strengths are in the range of low-density polyethylene (LDPE) [17] and are similar to poly(butylene sebacate) (PBSeb) [77], a polyester bioplastic with a tensile strength of 2.6 MPa. By comparison, previously reported hemicellulose benzoate properties relied on composites with 50% poly(vinyl alcohol) (PVOH) to provide strength and elasticity [78]. These results establish a robust proof-of-concept for wood-derived aromatic bioplastics, providing a foundation for future ductility optimization strategies.

## 4. Conclusions

This study demonstrates the effectiveness of the TFAA impelling agent method for synthesizing benzoate and cinnamate esters from cellulose and pine sawdust, transforming woody residues into high-performance thermoplastic bioplastics. Contrary to previous work, our results reveal that pine esters consistently outperform pure cellulose in terms of mechanical properties, reaching tensile strengths of 5 to 8 MPa. Simultaneously, these materials provide stable hydrophobicity with water contact angles ranging between 80° and 100°. This exhaustive characterization, which includes the first detailed mechanical testing of lignocellulose cinnamates, proves that the complex structure of wood can be harnessed to produce functional and sustainable materials.

The major novelty of this work lies in promoting cinnamates as a “greener” and technically superior alternative to benzoates, as cinnamic acid is less toxic, safer, and more readily bio-derived than its petrochemical counterpart [42,43,44]. The sustainability of the reaction using our method was additionally quantitatively demonstrated by the green metrics (PMI and E-factor). Benzoic acid can be obtained from natural sources such as gum benzoin from trees but most that is produced is from fossil sources. Cinnamic acid can be more readily obtained from natural sources [79]. Given their optimized thermal and mechanical properties, these aromatic esters are promising candidates for bio-based packaging applications. Future research will focus on validating their biodegradability and conducting a full Life Cycle Assessment (LCA), paving the way for the practical integration of these materials into a circular economy [80]. Finally, we aim to extend this method to other aromatic and unsaturated linear esters to broaden the range of available mechanical properties and meet the requirements of diverse industrial applications.

## Figures and Tables

**Table 1:** The layer spacing value of cellulose and pine derivatives from XRD analysis.

Sample	Layer Spacing, d (Å)
Pine Cinnamate	12.92
Pine Benzoate	13.43
MCC Cinnamate	11.19
MCC Benzoate	12.21

**Table 2:** The area of crystalline, area of amorphous and crystallinity index (CrI in %) of the celluloses in our samples using peak deconvolution and the values from the Segal method (%).

Sample	Crystalline	Amorphous	CrI (%)	Segal (%)
Pure MCC	1,076,298	856,499	55.7	76.0
Pine sawdust	499,512	503,425	49.8	55.9
MCC Benzoate	466,549	1,031,418	31.1	12.6
Pine Benzoate	349,364	540,347	39.3	5.8
MCC Cinnamate	699,761	1,098,829	38.9	12.2
Pine Cinnamate	566,829	851,283	40.0	10.3

**Table 3:** Thermal degradation, Td of native pine and MCC as well as the esterified products.

Sample	Main Thermal Degradation Range, Td (°C)	Tmax (°C)
Pine	313–370	357
MCC	333–368	358
Pine Benzoate	339–381	365
MCC Benzoate	333–376	360
Pine Cinnamate	346–383	367
MCC Cinnamate	342–376	361

**Table 4:** Glass transition temperature, Tg and softening temperature, Ts of modified pine and MCC.

Sample	Tg (°C)	Ts1 (°C)	Ts2 (°C)
Pine Benzoate	-	224	-
MCC Benzoate	279	227	274
Pine Cinnamate	151	151	-
MCC Cinnamate	137	137	254

**Table 5:** Processing windows based on the maximal and minimal temperatures.

Sample	Minimal Temperature	Maximal Temperature	Processing Window
Pine Benzoate	224	339	115
MCC Benzoate	279	345	66
Pine Cinnamate	151	346	195
MCC Cinnamate	137	342	205

**Table 6:** Mechanical properties of modified pine and MCC.

Sample	Tensile Strength (MPa)	Elongation at Break (%)	Young Modulus (GPa)
Pine Benzoate	5.47 ± 0.55	0.21 ± 0.16	3.43 ± 0.99
MCC Benzoate	1.27 ± 0.43	0.58 ± 0.42	0.64 ± 0.02
Pine Cinnamate	7.86 ± 2.76	0.38 ± 0.22	2.47 ± 0.25
MCC Cinnamate	2.63 ± 0.06	0.68 ± 0.23	1.02 ± 0.01

## Data Availability

The original contributions presented in this study are included in the article/Supplementary Materials. Further inquiries can be directed to the corresponding authors.

## Supplementary Materials

The following supporting information can be downloaded at: https://www.mdpi.com/article/10.3390/ma19030574/s1, Figure S1: Segal method to calculate crystallinity index; Figure S2: Diffraction pattern of MCC showing peak deconvolution; Figure S3: Diffraction pattern of pine showing peak deconvolution; Figure S4: Diffraction pattern of pine benzoate showing peak deconvolution; Figure S5: Diffraction pattern of pine cinnamate showing peak deconvolution; Figure S6: Diffraction pattern of MCC benzoate showing peak deconvolution; Figure S7: Diffraction pattern of MCC cinnamate showing peak deconvolution; Figure S8: Contact angles of water droplets on the surface of films over time.

## Abbreviations

The following abbreviations are used in this manuscript:

DSC	Differential Scanning Calorimetry
FTIR	Fourier Transform Infrared
LCA	Lifecycle Assessment
MCC	Microcrystalline Cellulose
NMR	Nuclear Magnetic Resonance
SEM	Scanning Electron Microscopy
TGA	Thermal Gravimetric Analysis
TMA	Thermal Mechanical Analysis
XRD	X-ray Diffraction
