# Supplementary material for: Aromatic Ester Bioplastics from Wood and Cellulose: Cinnamates as Greener Alternatives to Benzoates
Source: Materials (Basel). 2026 Feb 2;19(3):574. doi: 10.3390/ma19030574 (PMC12897698; doi:10.3390/ma19030574)
Supplement: Supplementary file 1 [file materials-19-00574-s001.zip › materials-4111546-supplementary.pdf]

Supporting Information:

Aromatic ester bioplastics from wood & cellulose: cinnamates as 'greener' alternatives to benzoates

Authors: Luke Froment, Jacqueline Lease, Firmin Obounou Akong, Prabu Satria Sejati, Christine Gérardin Charbonnier, Yoshito Ando*, Philippe Gérardin*

Addresses:
Luke Froment, Firmin Obounou Akong, Christine Gérardin Charbonnier and Philippe Gérardin: LERMAB, INRAE, Université de Lorraine, 54000 Nancy, France,

Jacqueline Lease: Department of Life Science and Systems, Engineering, Graduate School of Life Science and Systems Engineering, Kyushu Institute of Technology, Kitakyushu, Fukuoka 808-0196, Japan

Yoshito Ando: Collaborative Research Centre for Green Materials on Environmental Technology, Kyushu Institute of Technology, Kitakyushu, Fukuoka 808-0196, Japan

Prabu Satria Sejati: Research Center for Biomass and Bioproducts, National Research and Innovation Agency (BRIN), 16911 Bogor, Indonesia

e-mails of the corresponding authors: Philippe Gérardin philippe.gerardin@univ-lorraine.fr Yoshito Ando yando@life.kyutech.ac.jp

Orcid URLs : Luke Froment 0000-0002-6179-1964 Jacqueline Lease 0009-0006-6316-782X Prabu Satria Sejati 0000-0002-9961-7830 Christine Gérardin 0000-0003-3371-4061 Yoshito Ando 0000-0003-3839-0705 Philippe Gérardin 0000-0002-0911-0105

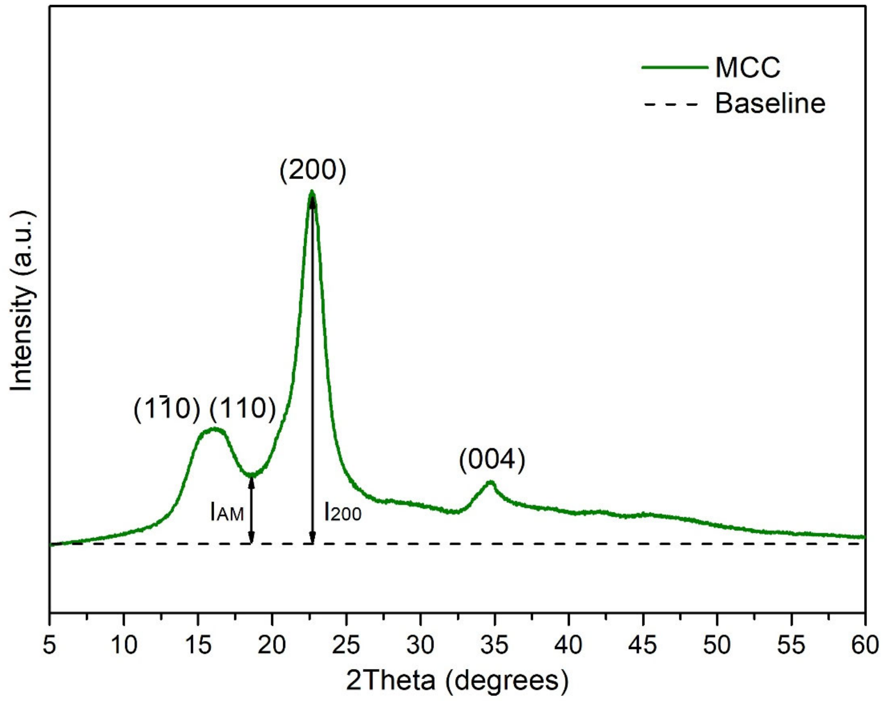

Figure S1 Segal method to calculate crystallinity index

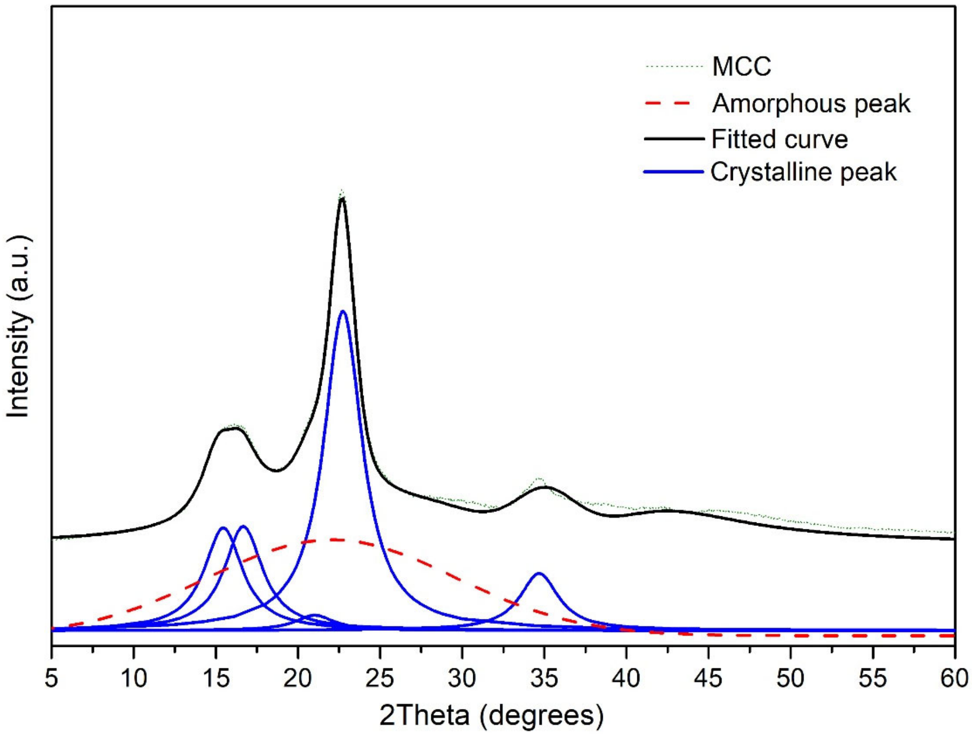

Figure S2 Diffraction pattern of MCC showing peak deconvolution

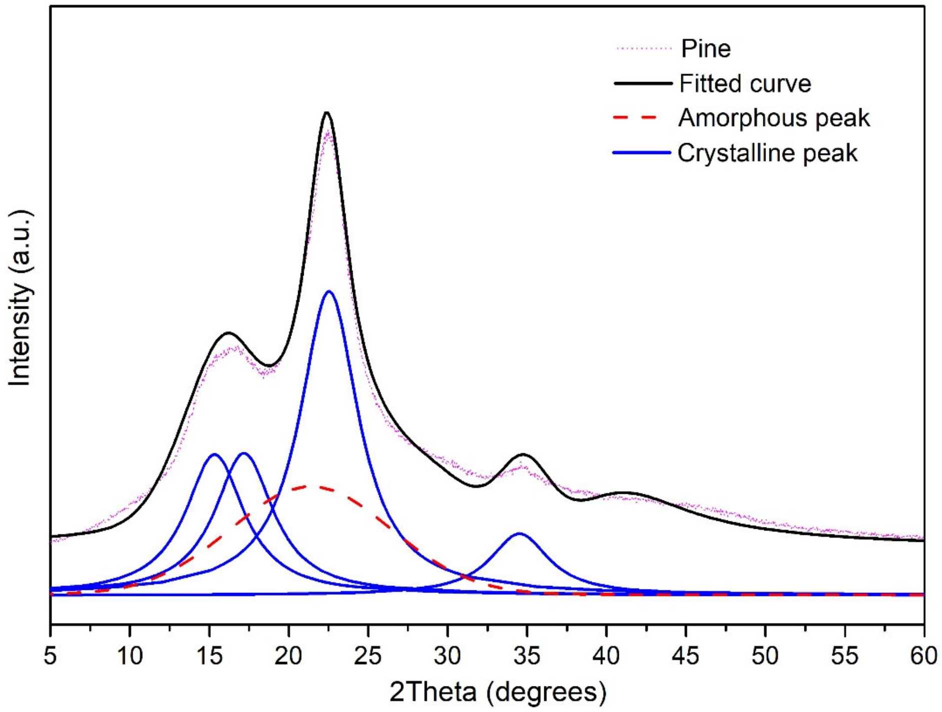

Figure S3 Diffraction pattern of pine showing peak deconvolution

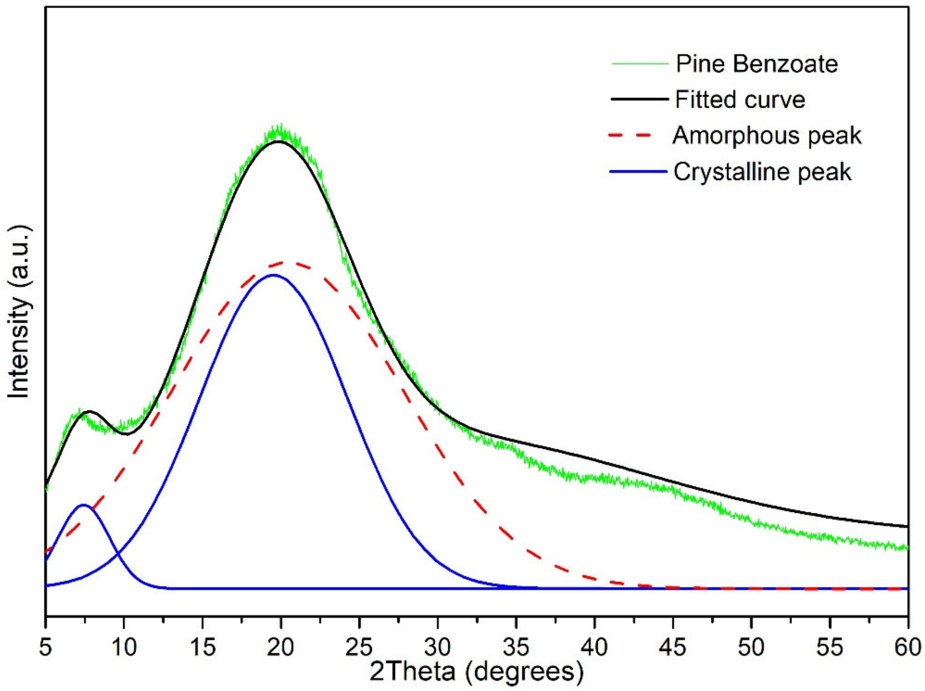

Figure S4 Diffraction pattern of pine benzoate showing peak deconvolution

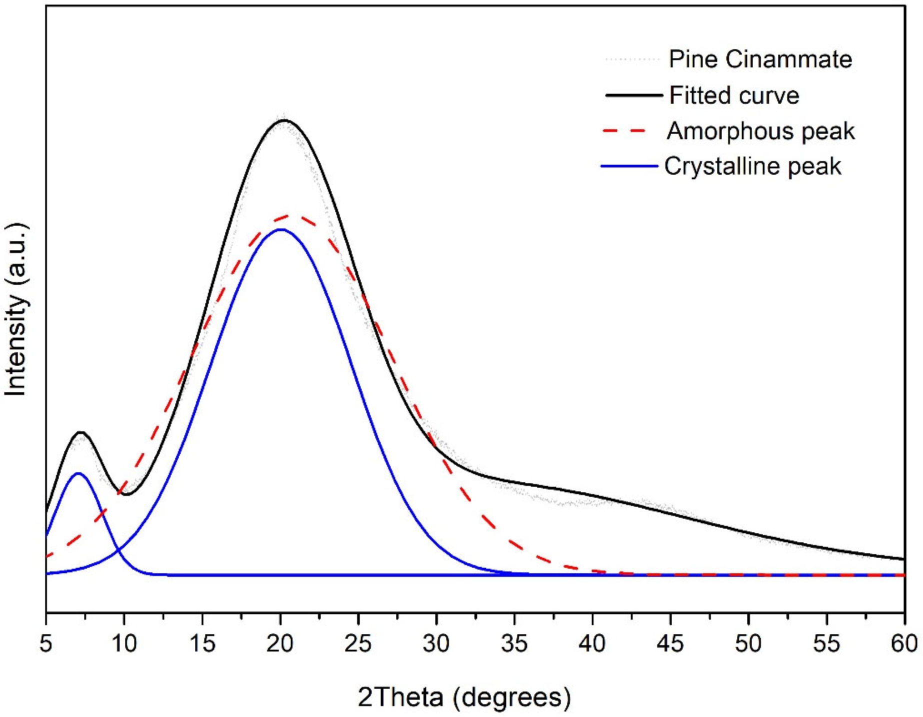

Figure S5 Diffraction pattern of pine cinnamate showing peak deconvolution

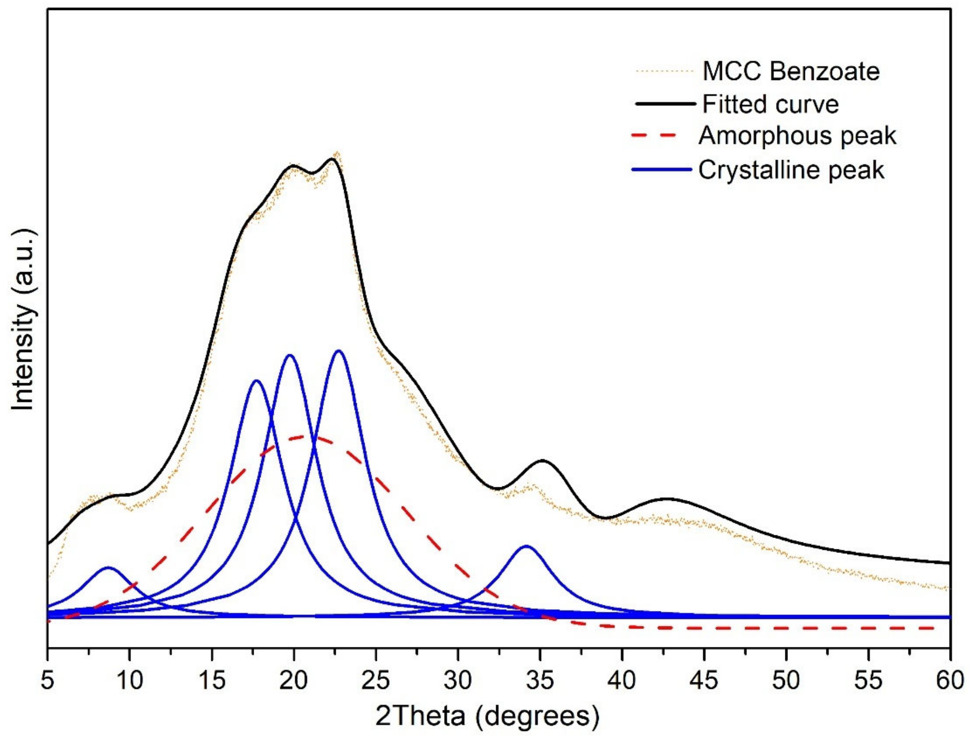

Figure S6 Diffraction pattern of MCC benzoate showing peak deconvolution

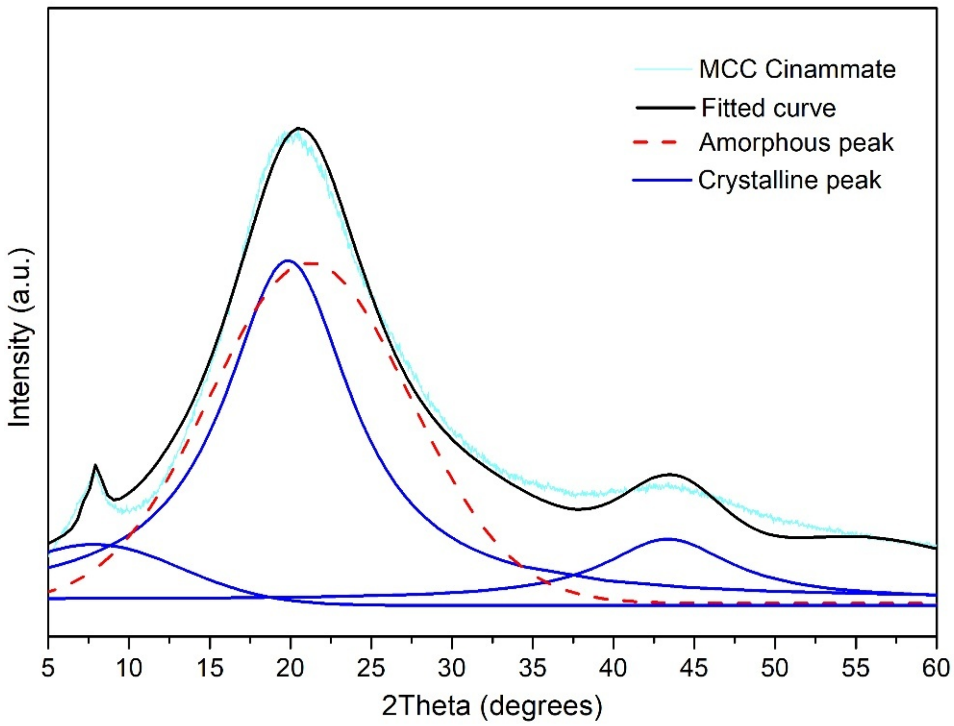

Figure S7 Diffraction pattern of MCC cinnamate showing peak deconvolution

Contact angle measurements were also performed using a Kruss DSA100S goniometer, with values in good agreement.

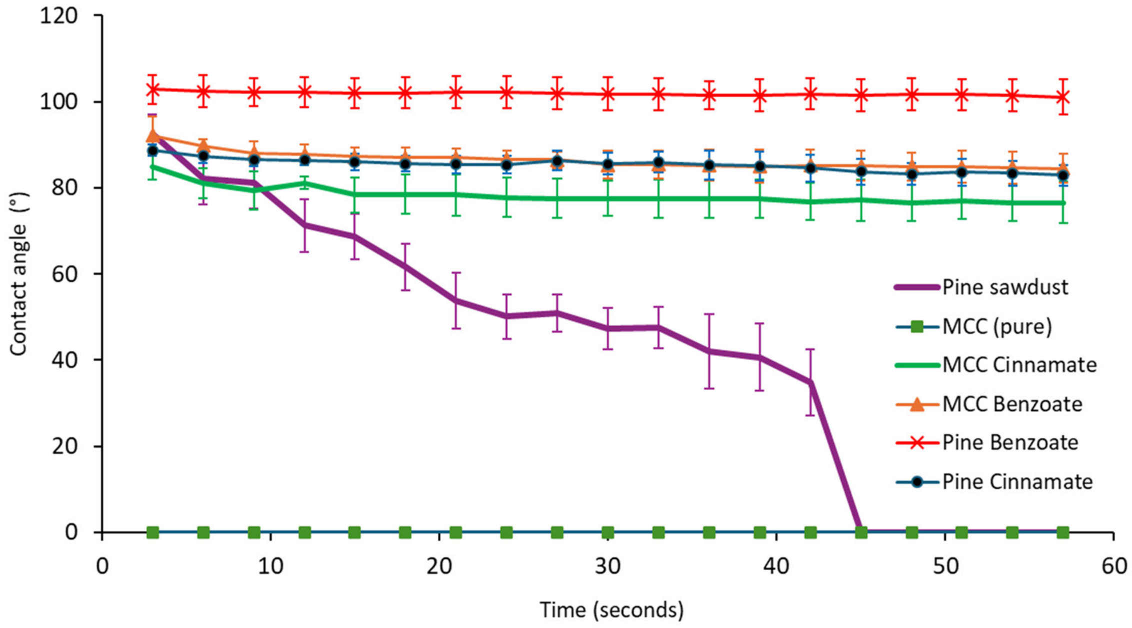

Figure S8 Contact angles of water droplets on the surface of films over time
